# Training on adequate use of opioid analgesics in West and Central Africa: a neglected step on the way to access to essential medicines?

**DOI:** 10.1186/s40545-021-00388-7

**Published:** 2021-12-09

**Authors:** Serena Frau, Anselme Mubeneshayi Kananga, Jackie Ndona Kingolo, Ghislaine Mbelu Kanyunyu, André Katele H. Zongwe, Aaron Nshindi Tshilengi, Raffaella Ravinetto

**Affiliations:** 1 Master in Tropical Medicine and Global Health, University of Florence, Florence, Italy; 2 Master in Tropical Medicine and Global Health, University of Brescia, Brescia, Italy; 3 International Youth Association for Development (IYAD), Antwerp, Belgium; 4 Plateforme Hospitalière de la République Démocratique Congo, Kinshasa, Democratic Republic of Congo; 5 Ministry of Health, ACOREP, Kinshasa, Democratic Republic of Congo; 6 University Hospital, University of Kinshasa, Kinshasa, Democratic Republic of Congo; 7 Palliafamilli, Kinshasa, Democratic Republic of Congo; 8 Department of Public Health, Institute of Tropical Medicine, Antwerp, Belgium

**Keywords:** Morphine, Opioids, Controlled substances, Training, Education, Access to medicines, Palliative care, Equity, Global health, West Africa, Central Africa

## Abstract

Alleviating acute and chronic pain is a moral imperative for health professionals and health systems, and it requires adequate access to and use of essential opioid analgesics. However, this is still a neglected issue in global health, with striking inequalities in opioids availability between high and low- and middle-income countries. Countries most affected by lack of access are those with a fragile political situation and weak regulatory and healthcare systems. The main threats to accessibility, availability and affordability are situated at different levels: legislation and policy, financing, knowledge and cultural behavior, erroneous beliefs, and training and education. Among these threats, the lack of (adequate) training and education seems to be a cross-cutting issue. Exploring the current body of knowledge about training and educational activities related to use of opioid analgesics and palliative care, is helpful to understand gaps and to delineate priorities for setting up adequate interventions. When applied to West and Central Africa, this exercise reveals that there is little information (easily) available in the public domain. The African Palliative Care Association (APCA) appears to be the leading provider of capacity building activities in this region for key stakeholders, including national authorities, healthcare professionals and the general population; it is also very active in publishing and communicating about these issues. However, apart from APCA, there is little information on training programs’ contents and long-term outcomes. Furthermore, trainings rarely target important stakeholders such as lawmakers, regulators, supply officers and the lay public (i.e., patients, caregivers, community leaders and members of the society as a whole). Hence, it is urgent to fill the existing gaps in training and educational activities to improve access to essential opioid analgesics in West and Central Africa, involving different stakeholders at the national and regional level. Furthermore, such experiences should be published and made publicly available to allow for mutual learning and further upscale.

**Supplementary Information:**

The online version contains supplementary material available at 10.1186/s40545-021-00388-7.

## Introduction

The gap in access to opioid analgesics between high-income countries and low- and middle-income countries (LMIC) is still huge [1–5]. In 2019, the total consumption of opioid analgesics for medical and scientific purposes, expressed as defined daily doses for statistical purposes (S-DDD) per million inhabitants, was 9200 S-DDD in Western Europe versus 90 S-DDD in Africa, and 20 S-DDD in South Asia [2]. It was also estimated that the 92% of morphine was consumed in countries where the 17% of the world population lives [1].

Morphine, methadone, hydromorphone and oxycodone (for adults and children), and codeine and fentanyl (for adults only) are listed in the WHO Model Lists of Essential Medicines [6, 7], and they are controlled substances under the international Single Convention on Narcotic Drugs [8]. International regulations aim at a balance between medical availability, and the prevention of abuse and diversion [1, 2]. But many barriers to access remain at the various levels, i.e.  legislation and policy, financing, knowledge, cultural attitude, training and education [3, 4, 9, 10]. In particular, an integrated educational approach addressing key stakeholders across policymaking, regulation, health systems, and communities, would help to upscale access [10, 11], as reiterated in the United Nations Office on Drugs and Crime (UNODC) Resolution 63/3 in 2020 [12].

## The case of West and Central Africa

We hypothesized that educational needs are unlikely to be adequately met in fragile health systems, characterized by a dearth of health workers with adequate experience in opioid prescription and use. Therefore, in February 2021, we conducted an exploratory, non-systematic literature review of training and educational programs related to use of opioid analgesics and palliative care, and conducted in the 24 countries in West and Central Africa, i.e., Benin, Burkina Faso, Cabo Verde, Cameroon, Central Africa Republic, Chad, Democratic Republic of Congo (DRC), Republic of Congo, Côte d'Ivoire, Equatorial Guinea, Gabon, Ghana, Guinea Bissau, Guinea, Liberia, Mali, Mauritania, Niger, Nigeria, Sao Tome and Principe, Senegal, Sierra Leone, The Gambia, and Togo. We integrated the PubMed search with first-hand information from the Democratic Republic of Congo (DRC), and with documents from the websites of three international organizations (i.e., the African Palliative Care Association (APCA), the Worldwide Hospice Palliative Care Alliance and the International Association for Hospice and Palliative Care) and 11 national palliative care associations and teaching hospitals (details of the search methodology are in Additional file 1).

### S*upply*

The Single Convention on Narcotic Drugs stipulates that countries must submit to the International Narcotics Control Board (INCB) information on the consumption of opioid analgesics during the past year, and on the estimated needs for next year, as a pre-requisite for procurement [1, 2]. Noteworthy, INCB considers a consumption between 100 and 200 S-DDD as inadequate, and below 100 S-DDD as very inadequate [2]. Unfortunately, many countries seem to underestimate their actual needs [1]. For instance, only 11 out of the 24 countries in our sample submitted estimates in 2018. Furthermore, the submitted estimates look very low [13]: for the period 2015–2017, only Senegal declared a consumption above 200 S-DDD (215 S-DDD) [13], while all other countries declared consumptions below 48 S-DDD [13]. Inaccurate estimates can cause under-supply, distortion of demand, inappropriate prescriptions, and avoidable human suffering [1, 9]. To improve reporting, those in charge should be adequately trained for gaining the expertise to monitor consumptions, estimate needs, and navigate the national and international legislation. Furthermore, well-trained staff should be in adequate number, and well-resourced. Unfortunately, there seems to be little training for stakeholders in the procurement and supply chain. We only found an APCA *Continuing Medical Education* program to improve data collection systems in Ghana [14].

### Local production

To improve morphine availability in the public sector, Cameroon, Sierra Leone, Ghana, Nigeria, and Togo promote local production [14–16]. In Ghana, Togo and Nigeria, APCA provided training on the selection of formulations, access to raw materials, production, quality assurance, distribution and monitoring of consumptions [14]. In Togo, the government funded a local morphine production unit and provided relevant training for 18 staff [14, 15]. In Nigeria, the “*Treat the Pain*” collaboration empowered several teaching hospitals to start production between 2012 and 2013 [16, 17] Other initiatives may remain undocumented in the public domain. For instance, in DRC a pain relief NGO promoted a public–private initiative for producing oral morphine in 2015 (regrettably, the project had to be discontinued in 2019, but a new initiative is planned in collaboration with Kinshasa University—personal communication). Although local manufacturing can sustainably improve access, we did not find (easily accessible) information on the typology of manufacturing (i.e., industrial or compounding), on the contents of related training (apart from mention of a workshop on Good Manufacturing Practice in Nigeria [14]), and on continuous education. It is unclear if any lessons learned or recommendations could be extended to other settings.

### Palliative care

An important barrier to access to essential opioid analgesics is a weak, underdeveloped palliative care system [18, 19]. Across 24 countries, we found a clear description of palliative care provisions—even if not fully integrated in national health systems—only for The Gambia and Côte d’Ivoire [19]. There seem to be isolated initiatives for 11 countries, while for the remaining ones we found no published information [19]. The lack of national policies to develop palliative care services seems to correlate with dearth of palliative care research, resulting in poor visibility of needs, underfunding, and lack of opportunities for policy changes [20].

The training of healthcare professionals can reduce misconceptions and inadequate prescription [4, 9, 10, 20] but formal curricula seem to be scarce [18]. According to APCA,  Côte d’Ivoire, Ghana, Guinea, Niger, Nigeria and The Gambia have mandatory training course in palliative care in medical schools; and Côte d’Ivoire, Ghana, Guinea, Nigeria and The Gambia, in nursing schools [18]. However, we did not scrutinize public or private University’s websites. Furthermore, there may be unpublished experiences. For instance, a pilot training program in palliative care has been started in 2018 at the Institut Supérieur des Techniques Médicales (ISTM) in DRC, in collaboration with Palliafamilli.

Few published studies look at the impact of training. In Ghana and Nigeria, a training program improved doctors’ knowledge on appropriate use, opioids titration, and managing side effects [21]. The evaluation of a training program targeting 715 healthcare staff in 15 tertiary hospitals in Nigeria [22], indicates that participants enhanced their knowledge and reduced misconceptions on opioids use and abuse risk, leading to an increase in morphine use of 60.4% [22]. However, these experiences are limited to hospital settings; their impact on quality of care was not evaluated in the long-term; and we did not find information on the training contents, nor on lessons learned or recommendations for other settings.

### Who is being missed?

Lawmakers, regulators, and pharmaceutical supply officers have a crucial role in assessing needs and consumptions of medicines for pain control, and making them available. However, training programs addressing these stakeholders seem scarce or not structured in West and Central Africa. According to APCA, law enforcement officials received training in The Gambia [15], but we did not find details on contents and outcomes. Furthermore, patients, caregivers, community leaders and members of the society as a whole could play a key role in advocating for access to essential opioid analgesics for medical reasons, reducing stigma, and reshaping attitudes towards palliative care [4]. Unfortunately, this group seems neglected by training and awareness-raising activities, excluding some sporadic activities promoted by the APCA in six out of 24 countries [14].

## A call for inclusive training

The main limitation of this short report is its reliance on information available in peer-reviewed literature and in the websites of selected associations. Our first-hand information from DRC indicates that much more initiatives may exist. For instance, two training on opioids use were organized in DRC in 2018 and 2021 by the International Youth Association for Development and Palliafamilli (personal communication). However, initiatives that are not (yet) published or not easily retrievable will not be known to peers, and will miss the opportunity to inform and orient similar undertakings.

Integrated training activities remain insufficient, or insufficiently known, in West and Central Africa. The few documented experiences mainly focus on prescribers; are designed as short-term experiences; and the lack of (public) information on contents makes it difficult to share and widely apply good practices. Meanwhile, more needs are emerging due to severe COVID-19 [23, 24]. The seventy-third World Health Assembly recommended palliative care as a core component within the COVID-19 response plans [23], but to the best of our knowledge, palliative care and opioids management are generally not included in COVID-19 training of health staff in sub-Saharan Africa [24]. This may reflect the observed “lacunae in the prevailing ideologies of global health”, which prioritize treatment and eradication of diseases, while the need for pain management may be perceived as a mark of failure and a drain of resources [5].

## Conclusions

Training is an important component of an improved policy approach to pain control. Some short-term measures could perhaps be easily implemented in West and Central Africa. For instance, INCB e-learning modules addressing competent authority’s needs [2] could be translated and further disseminated; and publicly sharing the contents and impact of other experiences, would promote mutual learning and the development of common approaches. Access to opioid analgesics is a multidisciplinary undertaking, thus trainings should address all those involved in regulation, supply, prescription, and use, from lawmakers to stakeholders in pharmaceutical systems, from prescribers to patients and communities. And they should be framed in the lens of equity in health and Universal Health Coverage.

## Supplementary Information

**Additional file 1.** Master thesis - Training programs to improve access to opioid analgesics for medical use: a review in Western and Central Africa.

## Data Availability

The internal report that inspired this manuscript is provided as Additional file 1: “Training programs to improve access to opioid analgesics for medical use: a review in Western and Central Africa”. Master in Tropical Medicine and Global Health. University of Florence, University of Brescia and Ospedale Sacro Cuore—Don Calabria di Negrar (Verona) Italy. 2019/2020.

## Abbreviations

APCA: African Palliative Care Association
DRC: Democratic Republic of Congo
INCB: International Narcotics Control Board
LMIC: Low- and middle-income countries
S-DDD: Defined daily doses for statistical purposes
WHO: World Health Organization
UNODC: United Nations Office on Drugs and Crime
