# Supplementary material for: Training on adequate use of opioid analgesics in West and Central Africa: a neglected step on the way to access to essential medicines?
Source: J Pharm Policy Pract. 2021 Dec 9;14:104. doi: 10.1186/s40545-021-00388-7 (PMC8655326; doi:10.1186/s40545-021-00388-7)
Supplement: Supplementary file 1 — Additional file 1. Master thesis - Training programs to improve access to opioid analgesics for medical use: a review in Western and Central Africa. [file 40545_2021_388_MOESM1_ESM.pdf]

Università degli Studi di Firenze
Università degli Studi di Brescia
Ospedale Sacro Cuore - Don Calabria di Negrar (Verona)

MASTER INTERUNIVERSITARIO DI I° LIVELLO IN
"MEDICINA TROPICALE E SALUTE GLOBALE"

Training programs to improve access to opioid analgesics for medical use: a review in Western and Central Africa

Relatore
Dr. Raffaella Ravinetto

Corsista
Serena Frau

A.A. 2019/2020

Index

Abstract 4
Background 5
Objective 7
Methods 7
Results 8
Country characteristics 9
Availability of opioid analgesics 14
Knowledge, training, and awareness 18
Educational interventions on palliative care and opioid analgesics 19
a) National authorities 19
b) Healthcare professionals 20
c) Other stakeholders (medical and non medical) 23
d) Patients and civil society 24
Discussion 25
Conclusion 28
References 29

"[...] The enjoyment of the highest attainable standard of health is one of the fundamental rights of every human being without distinction of race, religion, political belief, economic or social condition. [...]" (WHO Constitution 1948)

"Recognizing that the medical use of narcotic drugs continues to be indispensable for the relief of pain and suffering and that adequate provision must be made to ensure the availability of narcotic drugs for such purposes." (Single Convention on Narcotic Drugs 1961)

Abstract

Functioning palliative care services with access to opioid analgesics are essential for achieving Universal Health Coverage. Yet, the access gap between high-income countries and low and middle-income countries (LMICs) is huge. In 2019, the 75% of the world population, mainly living in LMICs, has limited or no access to proper pain relief. Barriers situate at different levels, i.e.: legislation/policy, financing, knowledge/cultural attitude and education/training. A 2020 Resolution of the United Nations Office on Drugs and Crime reiterated the need for educational interventions to improve access to essential opioids in LMICs. We investigated the characteristics of educational programs in 24 Western and Central African countries.

Methods We performed a PubMed search on palliative care and opioid analgesics policy, knowledge, training, and management, and we integrated this search with policy documents published by national and international palliative care organisations.

Results In our sample of 24 countries, few training programs have been identified: 12 out of 24 countries benefitted from palliative care training activities provided by the African Palliative Care Association and/or national palliative care associations. Palliative care is part of medical school curricula in 6 countries and of nursing school curricula in 5. Training activities explicitly include the topic of access to opioids in 5 countries. Hence, morphine is available in the public health sector in 11 countries, 5 of which compounding it locally. However, its annual consumption in the area results far below 1 mg/per capita/year, versus 6.48 mg/per capita/year worldwide. Among the 28 papers we retained for our research, only 7 evaluated the training impact on opioid analgesics use, referring to 4 countries only (Nigeria, Ghana, Cameroon, Sierra Leone). No long-term outcomes have been found.

Conclusion Training and awareness actions appear to be limited, scattered and poorly coordinated, thus insufficient to overcome the barriers to access. Western and Central African countries need to develop training activities explicitly addressing the procurement of/access to essential opioid analgesics to facilitate access to palliative care and pain management.

Background

Access to opioid analgesics is a highly neglected theme among the global health community. Despite the increasing burden of disease from pain, the world population is still facing an unequal availability of these medicines[1-4].

In 2019, the International Narcotics Control Board (INCB) estimated that 92% of available morphine for medical use was consumed in countries in which only 17% of the world population lived (United States of America, Canada, countries in Western Europe, Australia and New Zealand)[1]. On the contrary, the 75% of the world population, mainly living in low and middle-income countries (LMICs), has limited or no access to proper pain relief[1]. These figures also do not fit with the increasing burden in LMICs of non-communicable diseases and end-stage HIV/AIDS, all of which require treatments for moderate to severe pain[2,3]. It is estimated that more than 61 million people are suffering from life-threatening or life-limiting health conditions worldwide, and more than 80% of these patients live in LMICs with limited access to palliative care medicines[4].

A 2014 World Health Assembly Resolution (WHA)[5] gave the World Health Organization (WHO) the mandate to leading global stewardship for achieving universal access to palliative care as part of the universal health coverage. It reiterated three critical points:

- pain relief is a human right, and palliative care is fundamental to improving the quality of life, well-being, comfort and human dignity for individuals;
- access to palliative care is a fundamental component of the right to attain the highest standard of health and well-being; and
- palliative care should be included in the definition and context of universal health coverage.

However, the under-treatment of pain is still a significant challenge in most LMICs, with children being disproportionately impacted[4].

To explain unavailability, inaccessibility and/or unaffordability of narcotic medicines for pain control, several barriers have been identified, all linked to the complexity of this matter, such as: a) legislative and policy barriers (i.e.: problems in sourcing, onerous regulations, trade control measures); b) economic barriers (i.e.: pricing policy, limited national resources); and c) knowledge and cultural attitude (i.e.: lack of training/awareness of professionals, fear of diversion or prosecution, fear of addiction)[3,4,6-10].

In this work, we did not consider the impact of Covid-19 pandemic on access to controlled medicines, despite knowing that the Covid-19 is a further barrier on medicines availability due to the disruptions of the medicines supply chain. Moreover, it is an additional health condition that increases the controlled medicines needs worldwide.

a) Legislative and policy barriers

Since 1961, the Single Convention on Narcotic Drugs as amended by the 1972 Protocol, aims at achieving a balance between protecting societies from the misuse of narcotics medicines; preventing the abuse at individual level; and guaranteeing their availability for medical use, recognizing their essential role for pain relief[11]. The

Single Convention on Narcotic Drugs includes and regulates 136 active substances[11]; among them, 6 are listed in the WHO Model List of Essential Medicines, section 2.2 - opioid analgesics: specifically codeine, fentanyl, methadone, morphine, hydromorphone and oxycodone[12,13].
Nonetheless, this international regulation also represents an important barrier to access, mainly in countries with a weak pharmaceutical regulatory system and fragile health care system. The Convention[11], and other related international policies[14,15], governs the production, manufacture, distribution, storage, and consumption of controlled medicines, and stringently regulates the prescription and dispensing, as well as the import and export at international level. In fact, every country is bound to submit annually its estimates of narcotic medicines consumption and needs (quota by country) to the INCB, which evaluate the data and regulate the international trade between countries[11]. These mechanisms are meant to prevent and detect the misuse of opioids, but they also end up limiting access for legitimate medical use in countries with a lack of technical knowledge and resources to quantify the needs adequately, particularly in LMICs[8,16].
Additionally, national policy further limits the availability to controlled medicines in many countries, mainly through stringent regulations on distribution and prescription[8,16,17]. As a consequence, physicians have become accustomed to using inappropriate or inadequate substitutes for pain relief, and this creates a vicious circle, gives the misleading impression that there is no demand for opioid analgesics and wrongly justifies the low supply[6].

b) Economic barriers

The opioid analgesics listed in the WHO Model List of Essential Medicines[12,13] such as morphine and methadone, are available as cheap multi-source formulations. Nonetheless, some low-income countries still face economic obstacles to purchase them. Inadequate purchasing policies that rely on a single supplier, and complex procedures for obtaining licenses and import permits, raise the indirect costs and make these products unaffordable to some countries[3,8].

c) Knowledge and cultural attitude

The lack of education and awareness among healthcare professionals and health-workers seems one of the main impediments contributing to the underuse of opioids[6]. For instance, hesitation in prescribing due to the fear of creating dependence and to safety concerns for the patients appears as a recurring issue [3,6]. Requirements for specific licenses for prescribing and dispensing further reinforce the prescribers' hesitancy[6,8,18]. Additionally, the lack of awareness of patients, family members and caregivers about the availability of/right to pain management therapies, further "silence" the expression of needs, and thus restrains the access to adequate pain control and palliative care[3].
Finally, the lack of awareness and specific training for staff at national health authorities and regulatory authorities impedes enhancing a public health system focusing on palliative care and influences the evaluation of needs for opioid analgesics[6].

The ways forward

In this frame, a coordinated multi-sectorial approach appears to be a crucial step to build universal access to opioid analgesics and to answer patients' needs everywhere.

It has been suggested that education and awareness-raising activities are key-intervention, and that adequate strategies should include the education of all health system managers, health care staff including pharmacists, government officials, legal professionals and the police, as well as the broader civil society, e.g. media, spiritual leaders, human rights advocates, and the public[6,19].
Significantly, the 2020 Resolution 63/3 of the United Nations Office on Drugs and Crime (UNODC): *"Promoting awareness-raising, education and training as part of a comprehensive approach to ensuring access to and the availability of internationally controlled substances for medical and scientific purposes and improving their rational use",* reiterated the need of an inclusive system for educational interventions to improve access to essential opioids[20].
This seems particularly urgent for LMICs, and for countries with linguistic barriers to access the streamline body of scientific evidence and educational materials, mostly available in English only.

Objective

Based on the framework of the Resolution 63/3[20], we assessed the current body of knowledge on educational and awareness-raising programs in palliative care in Western and Central Africa to identify existing gaps in educational interventions and to tentatively suggest the design priorities for the setting-up of adequate interventions.

Methods

First, we listed the 24 LMICs in Western and Central Africa (box 1) based on the World Bank classification of national income[21], and we described and compared their socioeconomic context, i.e.: population density, Human Development Index (HDI), poverty index, health workers density etc. based on the datasets from the World Bank[22-24], the United Nations Development Programme[25], and the Institute for Health Metrics and Evaluation[26-28].

Box 1 – Countries included in the analysis

Benin	Democratic Republic of Congo (DRC)	Mauritania
Burkina Faso	Equatorial Guinea	Niger
Cabo Verde	Gabon	Nigeria
Cameroon	Ghana	Sao Tome and Principe
Central Africa Republic	Guinea Bissau	Senegal
Chad	Guinea	Sierra Leone
Congo	Liberia	The Gambia
Côte d'Ivoire	Mali	Togo

Second, we considered in our analysis the opioid analgesics included in:

- the 21st version of the WHO Model List of Essential Medicines List[12], and

- the 7th WHO Model List of Essential Medicines List for Children[13], and
- the National Essential Medicines List of the selected countries, when available.

Third, we conducted a literature research with PubMed to identify papers on educational interventions aiming to improve access to these medicines in the 24 selected countries. We searched for papers addressing in the main text:
- opioid analgesics policy, knowledge, training, and management; or
- palliative care policy, knowledge, training, and management.

We used the keywords: [palliative care]; [analgesics, opioid]; [education]; [country name]; [Africa, Western]; [Africa, Central]; [Africa], using subject headings and MeSH terms, without any language restriction or publication date limits (box 2).
We excluded modeling studies, papers focused on specific disease (i.e.: sickle cell disease) or procedures (i.e.: spinal anaesthesia), and studies referring to countries not included in our research.

Forth, we integrated the PubMed search by performing a non-systematic review of documents on training and education programs implemented in the Western and Central African countries, gathering data from the following sources: the African Palliative Care Association (APCA)[29]; the Worldwide Hospice Palliative Care Alliance (WHPCA)[30]; the International Association for Hospice and Palliative Care (IAHPC)[31] and national palliative care associations (if any).

Box 2 – PubMed research query (last update 18.02.2021)

(Western and Central Africa) AND (palliative care)
((Africa, Western [Mesh]) OR Africa, Central [Mesh]) AND (Palliative Care [Mesh])
((Africa, Western [Mesh]) OR Africa, Central [Mesh]) AND (analgesics, opioid [MeSH Terms])
(("africa, western"[MeSH Terms])) OR (africa, central[MeSH Terms])) AND (analgesics, opioid[MeSH Terms])
(Country name) AND (analgesics, opioid [MeSH Terms])
(Country name) AND (palliative care)
((analgesics, opioid[MeSH Terms]) AND (Education[MeSH Subheading])) AND africa
(((opioids[MeSH Subheading])) OR (analgesics, opioid[MeSH Terms])) AND (africa[MeSH Terms])
((analgesics, opioid[MeSH Terms]) AND (Education[MeSH Subheading])) AND africa
(((opioids[MeSH Subheading])) OR (analgesics, opioid[MeSH Terms])) AND (africa[MeSH Terms])

Results

The initial search in PubMed identified 672 articles from 21 Western and Central African counties. No paper has been found for Guinea Bissau, Guinea, and Sao Tome and Principe. After the first screening of title and abstract, 619 papers were excluded and 53 papers underwent a full-text review. 28 out of these 53 papers, referring to six countries only, met our inclusion criteria (figure 1): seven resulted about palliative care training, ten about palliative care knowledge, seven about palliative care policy and four about opioid analgesics management. Nigeria topped the list of the countries for number of articles (15), followed by Ghana (3), Democratic Republic of Congo (DRC) and Cameroon (2), Sierra Leone and Senegal (1). Four articles were not specific for a country but provided general information on palliative care policy. Eighteen out of 24 countries had no references in the literature review, meeting the inclusion criteria of our research.

Figure 1 – Flowchart of study selection

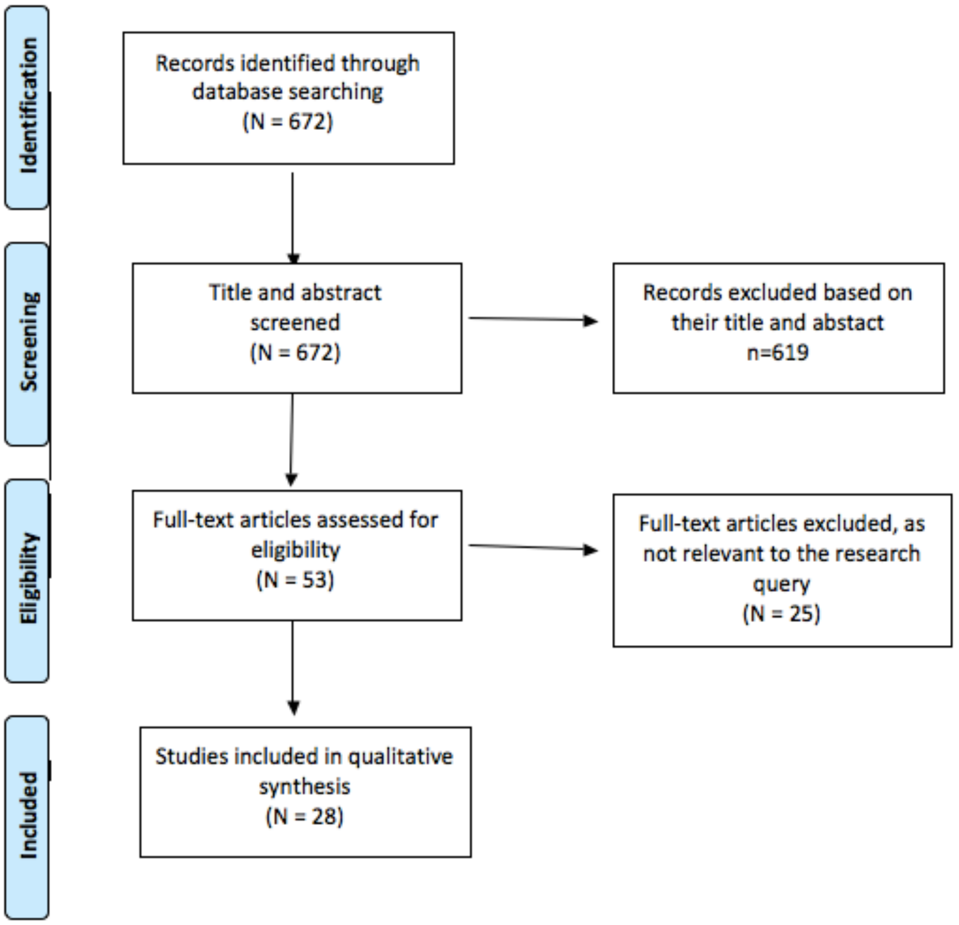

Further data on palliative care and opioid analgesics have been retained from the non-systematic review of published documents available in the websites of the APCA[29]; the WHPCA[30]; the IAHPC[31]. Additionally, we scrutinised the websites of 27 national palliative care association and teaching hospitals and, among them, 11 provided information on training and education.

Country characteristics

We compared the 24 LMICs in terms of socio-economic context (table 1), health service costs and personnel (table 2), and the palliative care facilities (tables 3 and 4).

Half of the countries are LICs (gross national income (GNI) per capita $1,035 or less), ten belong to the lower MIC group (GNI per capita in between $1,036 and $4,045). Only Equatorial Guinea and Gabon are classified as upper MIC (GNI per capita in between $4,046 and $12,535) (table 1).

Seventeen countries have a low Human Development Index (<0.546); among them, 13 register an international poverty rate (people living with less than $ 1.9) above 30%. Gabon is the only country with a high HDI (0.703) and it has the lowest international poverty rate in the area (3.4%).

Nigeria, the most populated country in Western and Central Africa counting about half of West Africa's population, is ranked 15th for HDI (low range), and 13th as an international poverty rate (39%).

Table 1 – Socio-economic context

Countries	Population, million	Life expectancy (2019)	Income group	GNI per capita, US$ (2019)	Human Development Index (2019)	International poverty rate
Benin	12.00	61.8	Lower MIC	1,250	0.545	49.6
Burkina Faso	20.3	61.6	LIC	780	0.452	43.8
Cabo Verde	0.6	73.0	Lower MIC	3,630	0.665	3.4
Cameroon	25.3	59.3	Lower MIC	1,500	0.563	26
Central Africa Republic	4.8	53.3	LIC	520	0.397	n/a
Chad	15.8	54.2	LIC	700	0.398	38.1
Congo	5.1	64.6	Lower MIC	1,720	0.574	38.2
Côte d'Ivoire	25.5	57.8	Lower MIC	2,290	0.538	29.8
Democratic Republic of the Congo (DRC)	86.7	60.7	LIC	530	0.480	77.2
Equatorial Guinea	1.4	58.7	Upper MIC	6,460	0.592	n/a
Gabon	1.9	66.5	Upper MIC	7,170	0.703	3.4
Ghana	30.1	64.1	Lower MIC	2,220	0.611	13
Guinea Bissau	2	58.3	LIC	820	0.480	68.4
Guinea	13.6	61.6	LIC	930	0.477	36.1
Liberia	5	64.1	LIC	580	0.480	44.4
Mali	19.7	59.3	LIC	870	0.434	50.3
Mauritania	4.5	64.9	Lower MIC	1,660	0.546	6
Niger	23.2	62.4	LIC	600	0.394	45.4
Nigeria	202	54.7	Lower MIC	2,030	0.539	39.1
Sao Tome and Principe	0.2	70.4	Lower MIC	1,930	0.625	35.6
Senegal	16.7	67.9	Lower MIC	1,460	0.512	38.5
Sierra Leone	7.9	54.7	LIC	540	0.452	43
The Gambia	2.4	62.1	LIC	750	0.496	10.3
Togo	8.2	61.0	LIC	690	0.515	51.1
Italy	61.0	83.5	HIC	34,530	0.9	1.4
Belgium	11.4	81.6	HIC	48,030	0.9	0.1

*Belgium and Italy have been included for comparison
n/a = not available
a. The World Bank in Western and Central Africa
b. The World Bank Macro Poverty Outlook for Sub-Saharan Africa
c. United Nations Development Programme - Human Development Reports
d. The World Bank - Poverty and Equity Database

Equatorial Guinea and Gabon have the highest total health spending per capita ($1,411 and $612, respectively), and the out-of-pocket spending[a32] represents about 20% of the total (still, such figures are significantly lower than those in HICs, as showed by the comparison with Belgium and Italy). In most other countries, the total health spending per capita is below $300, and the out-of-pocket spending[a32] represents more than 30%, reaching 70% in Nigeria (table 2).
Accordingly, the 2019 Universal Health Coverage (UHC) effective coverage index[b33] varied between 22.3% in Central Africa Republic to 62.2% in Cabo Verde, with only five countries registering a UHC index above 50%.

Western and Central Africa have traditionally faced a lack of skilled health workforce. In four out of 24 countries there is less than 1 doctor for 10,000 inhabitants and in 13 of them there is only one doctor for 10,000 inhabitants. In 17 out of 24 countries we can count less than 10 nurses for 10,000 people (table 2). Cabo Verde and Gabon seem to be the countries with more doctors per population (8:10,000 and 7:10,000, respectively), while Ghana and Gabon those with more nurses (42:10,000 and 29:10,000, respectively).

Table 2 - Healthcare services costs and personnel

Countries	Total health spending per capita (2015)[a]	Out of pocket (%)[a]	UHC effective coverage index[b]	Health workers (per 1,000)[c]	Physicians (per 1,000)[d]	Nurses and midwives (per 1,000)[d]
Benin	105.0	35.5	44.6	1.00	0.10	0.40
Burkina Faso	83.0	38.6	41.8	1.2	0.1	0.9
Cabo Verde	318.0	22.2	62.2	3	0.8	1.3
Cameroon	116.0	68.5	42.3	2.9	0.1	0.9
Central Africa Republic	35.0	34.2	22.3	0.5	0.1	0.2
Chad	89.0	37.2	31.4	0.8	0	0.2
Congo	312.0	17.4	43.9	1.6	0.2	0.6
Côte d'Ivoire	179.0	54.6	43	1.7	0.2	0.6
DRC	46.0	37.4	45.2	0.6	0.1	1.1
Equatorial Guinea	1,411.0	20.7	50	2.2	0.4	0.5
Gabon	612.0	22.0	53	2.1	0.7	2.9
Ghana	146.0	27.1	49.1	3.2	0.1	4.2
Guinea Bissau	101.0	34.5	35.7	1.3	0.1	0.7
Guinea	77.0	52.1	32.3	0.9	0.1	0.1
Liberia	345.0	7.8	47.6	1.7	0	0.5
Mali	162.0	43.6	40.7	0.9	0.1	0.4
Mauritania	153.0	44.7	53.3	1.5	0.2	0.9

a. *Out-of-pocket payments*: direct payments made by individuals to health care providers at the time of service use. This excludes any prepayment for health services, for example in the form of taxes or specific insurance premiums or contributions and, where possible, net of any reimbursements to the individual who made the payments[32].
b. *Universal Health Coverage effective coverage index*: aims to represent service coverage across population health needs and how much these services could contribute to improved health[33].

Niger	66.0	49.5	35	0.7	0	0.3
Nigeria	225.0	70.1	38.3	2.1	0.4	1.2
Sao Tome and Principe	251.0	11.9	54.8	2.1	0.1	1.9
Senegal	121.0	33.8	49.6	1.3	0.1	0.3
Sierra Leone	255.0	50.1	42.1	3	0	0.2
The Gambia	151.0	13.6	48.1	1.4	0.1	1.5
Togo	81.0	44.3	42.8	1.6	0.1	0.4
Italy	3,311.0	21.7	88.9	12.4	4	5.7
Belgium	4,751.0	17.8	87.3	17.3	3.1	19.5

*Belgium and Italy have been included for comparison

a. Financing Global Health 2016: Development Assistance, Public and Private Health Spending for the Pursuit of Universal Health Coverage

b. IHME Country Profiles

c. IHME. Health-related SDG

d. The World Bank Data - World Health Organization's Global Health Workforce Statistics

Palliative care services seem to be still insufficient in number and organisation (table 3)[34,35]. Based on publicly-available information referring to 21 out of 24 countries of our sample, there are no structured palliative care services in five countries (Burkina Faso, Central Africa Republic, Liberia, Mali, Sao Tome and Principe), while there is only one palliative care service per million of inhabitants in 13 of them. The small Gambia (2.4 million inhabitants) has the highest rate per population (5.2 palliative care services per million). No information has been found about Cabo Verde, Chad and Guinea Bissau.

Thirteen countries have a national palliative care plan. In six out of these 13 countries, it is a stand-alone program, while in the remaining seven it is part of the cancer or the HIV plan. Benin is the only country explicitly including the palliative care strategy into its national law.

Table 3 – Palliative care services

Countries	PC services	PC Services per Million	Stand-alone national PC plan	PC section in Cancer Plan or HIV Plan	PC in National Law [b]	National PC clinical guidelines	Ministry of Health - PC responsible	National Health budget
Burkina Faso	0	0		yes				
Central Africa Republic	0	0		yes				
Liberia	0	0					yes	
Mali	0	0		yes				
Sao Tome and Principe	0	0						
DRC	4	0.05					yes	
Cote d'Ivoire	2	0.09	yes [b]	yes		yes	yes	yes
Nigeria	17	0.09		yes			yes [b]	
Niger	2	0.1	yes [b]					
Ghana	4	0.15	yes [b]	yes				
Guinea	2	0.16	Yes	yes		yes	yes	
Benin	2	0.18	yes [b]		yes			

Congo	1	0.22						
Togo	2	0.27	yes [b]				yes	
Cameroon	7	0.3						
Senegal	5	0.33		yes			yes	
Mauritania	2	0.49						
Sierra Leone	4	0.62		yes				
Gabon	2	1.16						
Equatorial Guinea	1	1.18					yes	
The Gambia	10	5.2		yes		yes	yes	yes
Cabo Verde	n/a	n/a	n/a	n/a	n/a	n/a	n/a	n/a
Chad	n/a	n/a	n/a	n/a	n/a	n/a	n/a	n/a
Guinea Bissau	n/a	n/a	n/a	n/a	n/a	n/a	n/a	n/a

PC = palliative care; n/a = not available
a. APCA. Atlas of Palliative Care in Africa 2017
b. Clelland D et al. Palliative Medicine Reports 2020; 1: 1

In the G*lobal atlas of palliative care* published in 2020, the WHPCA reported the data on palliative care development in this area[7]. They showed that nearly half of the 24 countries in Western and Central Africa have some palliative care provisions, but neither well integrated nor supported by national policy, and/or heavily dependent on external donors' funding (i.e.: Nigeria[36]). Furthermore, they all rely on limited availability of morphine, and the number of palliative care services is small. The remaining 11 countries have no palliative care services established or not yet programmed (table 4)[7,35,37].

Côte d'Ivoire seems the most advanced country in palliative care development, having started integrating palliative care into the healthcare services[7]. Furthermore, it is reported that the collaboration between the Hope Worldwide charity[38] and the APCA pushed palliative care improvement, by increasing the availability of appropriate pain medicines, by developing a country-specific advocacy program, and by strengthening the national network[39].

Table 4 – WHPCA categorisation of palliative care development (2017)

**No Known PC activities**	Cabo Verde, Central Africa Republic, Chad, Congo, Guinea Bissau, Mali
**Capacity building PC activities planned, although no service yet established**	Burkina Faso, Equatorial Guinea, Gabon, Liberia, Sao Tome and Principe
**Isolated PC provision**	Benin, Cameroon, DRC, Ghana, Guinea, Mauritania, Niger, Nigeria, Senegal, Sierra Leone, Togo
**Generalised PC provision**	The Gambia
**PC at preliminary stage of integration**	Côte d'Ivoire

PC = palliative care
a. WHPCA. Global Atlas of Palliative Care - 2nd Edition
b. Clelland D et al. Palliative Medicine Reports 2020, 1: 1
c. Clark D et al. J Pain Symptom Manage 2020; 59: 794e807

Availability of opioid analgesics

The opioid analgesics listed in the 21st version of the WHO Model List of Essential Medicines[12] and in the 7th WHO Model List of Essential Medicines for Children[13] (section 2.2) are: morphine (immediate-release and slow-release tablets and granules, oral solution and injection) and methadone (tablet, oral solution), both for adults and children; fentanyl (patches) and codeine (tablets) for adults only. As for morphine's alternative, WHO considers hydromorphone and oxycodone. All these medicines are controlled and regulated by the Single Convention on Narcotic Drugs[11].

Among the 24 countries included in this research, 14 have published the national list of essential medicines. All these 14 countries included morphine among the essential opioid analgesics, 9 of them fentanyl and/or its analogues (alfentanil, sufentanil or remfentanil), 7 of them codeine or dihydrocodeine, and only 1 of them included methadone (table 5).
Additionally, 12 out of the14 countries with the national list of essential medicines (excluding Chad and Senegal) integrated the WHO Model List with other opioid analgesics, including: pethidine (medicine controlled under the Single Convention on Narcotic Drugs[11]); buprenorphine and pentazocine (which are controlled under the Convention on Psychotropic Substances of 1971[14]); and nalbuphine and tramadol which instead are not under international control and have no international restrictions (table 5).

Table 5 - WHO and National List of Essential Medicines

	International regulation on controlled medicines	WHO EML - Opioid analgesics	National EML - Opioid analgesics
**Morphine**	**Single Convention on Narcotic Drugs 1961 (Schedule I)**	x	14/14 Benin, Burkina Faso, Cameroon, Central African Republic, Chad, Congo, DRC, Ghana, Liberia, Mali, Nigeria, Senegal, Sierra Leone, The Gambia
**Hydromorphone Oxycodone**	**Single Convention on Narcotic Drugs 1961 (Schedule I)**	As alternatives to morphine	
**Codeine**	**Single Convention on Narcotic Drugs 1961 (Schedule II)**	x	6/14 Benin, Burkina Faso, Central African Republic, Chad, Congo, DRC
**Dihydrocodeine**	**Single Convention on Narcotic Drugs 1961 (Schedule II)**		1/14 Nigeria
**Methadone**	**Single Convention on Narcotic Drugs 1961 (Schedule I)**	x	1/14 Benin
**Fentalyl**	**Single Convention on Narcotic Drugs 1961 (Schedule I)**	x	8/14 Benin, Chad, Congo, DRC, Ghana, Mali, Nigeria, The Gambia
**Alfentanil Remfentalil Sulfentanil**	**Single Convention on Narcotic Drugs 1961 (Schedule I)**		3/14 Benin, Congo, Senegal
**Pethidine**	**Single Convention on Narcotic Drugs 1961 (Schedule I)**		7/14 Cameroon, Central African Republic, DRC, Ghana, Liberia, Nigeria, The Gambia
**Buprenorphine**	**Convention on Psychotropic Substances of 1971 (Schedule III)**		6/14 Benin, Burkina Faso, Cameroon, Congo, Mali, Nigeria

**Pentazocine**	**Convention on Psychotropic Substances of 1971 (Schedule III)**		5/14 Central African Republic, DRC, Nigeria, Sierra Leone, The Gambia
**Nalbuphine***	**Not Controlled**		2/14 Benin, The Gambia
**Tramadol***	**Not Controlled**		4/14 Cameroon, Congo, Sierra Leone, The Gambia

* Tramadol and nalbuphine are not under international control; therefore there are no international restriction for their purchase.

An accurate estimation of the needs of these medicines in the countries included in our sample appears difficult, particularly for those included in the Single Convention on Narcotic Drugs of 1961 (which are, in our sample: codeine, dihydrocodeine, fentanyl and its analogues, hydromorphone, methadone, morphine, oxycodone and pethidine). As stipulated by the Single Convention on Narcotic Drugs of 1961[11], all countries have to submit the statistical information on controlled narcotic medicines used during each year, as well as the estimate needs for the following year (the "quota") to the INCB. Without the submission, importation could be refused.

Unfortunately, this is a time- and resource-demanding exercise, and 13 out of 24 countries delivered no data on consumption for the period 2015-2017[40]. More recently, 16 countries presented a quota for 2021, while for 8 of them (Central African Republic, Chad, Congo, Equatorial Guinea, The Gambia, Guinea-Bissau, Niger, Sao Tome and Principe) the annual estimates were established by the INCB (table 6)[41].

In 2018, the total use of opioid analgesics, calculated as defined daily doses for statistical purposes (S-DDD)[c] per million inhabitants per day, was of 8,812 S-DDD in Europe, while it was only of 174 S-DDD in Africa[40]. The cut-off used by INCB for adequate availability is 200 S-DDD. More in details, the INCB considers levels of consumption of controlled opioid analgesics in quantities between 100 and 200 S-DDD to be inadequate, and less than 100 to be very inadequate[42].
Among the 11 countries included in our sample which deliver their data on consumption, Senegal reported the highest average consumption of opioid analgesics in the timeframe 2015–2017 (215 S-DDD), followed by Cabo Verde (48 S-DDD), Ghana (23 S-DDD) and Benin (10 S-DDD). Worldwide, the United States was the country reporting the highest use (32,394 S-DDD)[40].

Similarly, morphine consumption was 384 S-DDD in Europe, compared to 27 S-DDD in Africa[40]. Moving to our sample of countries, in 2014 in all but one country (Sao Tome and Principe) the overall morphine consumption was below 0.1 mg/per capita/year[40] vs. 6.48 mg/per capita/year worldwide[43]. If we compare the data of 2014[34] with those of 2017[40], only Senegal increased its use (from 0.14 to 2.40 mg/per capita/year) still keeping far below the average worldwide use of 6.48 mg/per capita/year. Morphine represented the 30% of the total opioid consumption in the period 2015–2017 in eight out of 11 countries.

---

c. *Defined daily doses for statistical purposes* (S-DDD): technical units of measurement for the purpose of statistical analysis and are not recommended prescription doses. It replaced the term "defined daily doses", which is the assumed average maintenance dose per day for a drug used for its main indication in adults. Certain narcotic drugs may be used in certain countries for different treatments or in accordance with different medical practices and, therefore, a different daily dose could be more appropriate[40].

For 2021, the self-reported morphine quota is above 0.1 mg/per capita/year only for Central Africa Republic, Ghana, Liberia, Mauritania and Senegal[41]. In 14 out of 24 countries morphine covers more than 25% of the demand.

Table 6 – Opioid analgesics consumption and quota

	Opioids* S-DDD/Million inhabit/day (2015-2017)	Morphine S-DDD/Million inhabit/day (2015-2017)	% Morphine on the total	Morphine per capita (mg/capita /year) 2017	Opioid** - Quota 2021 (kg)	Morphine - Quota 2021 (kg)	% Morphine on total quota
Nigeria	less than 1	less than 1	100	0.00	4438.4	66	1.5
DRC	1	1	100	0.01	130.1	40	30.7
Côte d'Ivoire	3	less than 1	33.3	0.04	122.1	2	1.6
Burkina Faso	3	less than 1	33.3	0.05	3.9	3.841	98.5
Chad	1	less than 1	100	0.06	1.7#	1	58.8
Ghana	23	3	13	0.07	352.9	80	22.7
Togo	4	less than 1	25	0.12	13.0	2	15.4
Sierra Leone	less than 1	less than 1	100	0.13	18.0	5	27.8
Benin	10	3	30	0.17	6.1	2	32.8
Cabo Verde	48	10	20.8	1.67	0.5	0.28	56
Senegal	215	73	34	2.40	1375.2	60	4.4
Equatorial Guinea	n/a	n/a	n/a	n/a	1#	0	0.0
Gabon	n/a	n/a	n/a	n/a	87.8	0.326	0.4
The Gambia	n/a	n/a	n/a	n/a	2.1#	0.225	10.7
Cameroon	n/a	n/a	n/a	n/a	58.1	8	13.8
Central Africa Republic	n/a	n/a	n/a	n/a	26.5#	6	22.6
Congo	n/a	n/a	n/a	n/a	9.5#	2.5	26.3
Guinea Bissau	n/a	n/a	n/a	n/a	6.7#	1.8	26.9
Niger	n/a	n/a	n/a	n/a	47.1#	15	31.8
Liberia	n/a	n/a	n/a	n/a	12.0	5	41.7
Mali	n/a	n/a	n/a	n/a	20.5	8.755	42.7
Guinea	n/a	n/a	n/a	n/a	3.1	1.5	48.4
Sao Tome and Principe	n/a	n/a	n/a	n/a	0.3#	0.185	61.7
Mauritania	n/a	n/a	n/a	n/a	15.0	15	99.9
Italy	7,512	100	1	25.36	48,314.5	2,600	5%
Belgium	19,960	214	1	7.28	9,613.3	110	1.1%

Belgium and Italy have been included for comparison
n/a = not available. The statistical report on narcotic drugs had not been received by 1 November 2018
** Alfentanil, Codeine, Dextropropoxyphene, Dihydrocodeine, Diphenoxylate, Etorphine, Fentanyl, Hydromorphone, Methadone, Morphine, Oxycodone, Oxymorphone, Pethidine, Pholcodine, Remifentanil, Sufentanil, Thebaine, Tilidine
# Annual estimates were established by the INCB
a. International Narcotics Control Board (INCB) - Estimated World Requirements for 2019. Statistics for 2017
b. INCB - Estimated world requirements of narcotic drugs in grams for 2021 (February update)

The 2017 *Atlas of Palliative Care in Africa* examined the opioids availability and accessibility in Africa, focusing on immediate-release morphine as key indicator (table 7)[34]. Oral immediate-release and injectable morphine have been included among the essential palliative care medicines toolkit and oral morphine availability is one of the WHO global health indicators[4,34,44]. In only 11 out of 24 countries, immediate-release oral morphine seems to be available in the public health sector[34,45,46-49]. Five of these 11 countries hold a local manufacturing program, where oral morphine is compounded locally with imported morphine powder, water, and preservative[45,47-49].

Morphine prescription is restricted to specific medical specialties (i.e.: oncologists) in half of the countries. In Cameroon and Sierra Leone nurses with appropriate training can prescribe it[34]; in Ghana and Sierra Leone, pharmacists are allowed to prescription, despite not been allowed to discretionally correct or accept the prescription[46] (table 7). Côte d'Ivoire, Nigeria and Senegal had restrictive laws for opioid prescription writing, requiring special prescription forms. Five countries limited the amount of opioid analgesics which can be prescribed by a physician to 2 days in Ghana, one week in Côte d'Ivoire and Liberia, one month in Senegal and Nigeria. In 4 countries, opioids are dispensed by hospital pharmacies only (table 7).

Table 7 – Morphine availability and accessibility

Countries	Availability of immediate-release oral morphine in the public health sector	Physician prescription: restriction by law for specialists only	Nurses prescription	Pharmacist prescription	Restriction to receive treatment	Prescription form	Days of supply	Dispensing pharmacy
Benin	no	Restricted	no	-	-	-	-	-
Burkina Faso	yes	Not restricted	no	-	-	-	-	-
Cabo Verde	no	-	-	-	-	-	-	-
Cameroon	reconstituted oral morphine from imported powder	Not restricted	yes	-	-	-	-	-
Central Africa Republic	no	Restricted	no	-	-	-	-	-
Chad	no	-	-	-	-	-	-	-
Congo	no	Restricted	no	-	-	-	-	-
Côte d'Ivoire	yes	Not restricted	no	-	no	restrictive	7	any
DRC	no	Restricted	no	-	-	-	-	-
Equatorial Guinea	no	Restricted	no	-	-	-	-	-
Gabon	no	Restricted	no	-	-	not restictive	-	-
Ghana	reconstituted oral morphine from imported powder	Not restricted	no	in emergency	special authorisation	not restictive	2	any
Guinea Bissau	no	-	-	-	-	-	-	-
Guinea	no	Restricted	no	-	-	-	-	-
Liberia	no	Restricted	no		special authorisation	not restictive	7	hospital
Mali	yes	Not restricted	no	-	-	-	-	-
Mauritania	no	Restricted	no	-	-	-	-	-

Niger	yes	Not restricted	no	-	-	-	-	-
Nigeria	reconstituted oral morphine from imported powder	Restricted	no	-	no	mix	30	hospital
Sao Tome and Principe	no	Not restricted	no	-	-	-		hospital
Senegal	yes	Restricted	no	-	-	-	28	
Sierra Leone	reconstituted oral morphine from imported powder	Not restricted	yes	special authorisation	special authorisation	mix	On discreti on	hospital
The Gambia	yes	Restricted	no	-	-	-	-	-
Togo	reconstituted oral morphine from imported powder	Restricted	no	-	-	-	-	-

a. APCA. Atlas of Palliative Care in Africa 2017
b. Cleary J et al. Ann Oncol 2013; 24 (S11): xi14–xi23
c. O'Brien M et al. Lancet Oncol 2013; 14: e176-82
d. Global Health Observatory data repository - indicator views WHO, 2019
e. APCA. Summary of Achievement 2011-2020
b. APCA. Annual Report 2017-2018 – Building bridges.
c. APCA. Annual Report 2018-2019 - Towards Universal Health Coverage.

Knowledge, training, and awareness

The APCA appears being the leading provider for training activities in Western and Central Africa[29,47-50]. The APCA collaborates with state and non-state actors, including Ministries of Health, hospice and national palliative care associations, academic institutions, healthcare professionals, the media and the general public[29]. Its main objectives are increasing knowledge and awareness of palliative care among all stakeholders; providing evidence-based knowledge to healthcare professionals; and integrating palliative care into the national health systems[29,34]. During the 2011-2020 timeframe, the APCA offered capacity building programs to 10 out of 24 countries, and provided translated training materials for 7 other countries (Benin, Burkina Faso, Chad, Congo, Guinea, Mauritania and Niger)[47-50].

Besides the APCA, 27 national palliative care associations and teaching hospitals are active in 13 countries, 11 of them providing training activities (table 8).
No action on palliative care education has been identified in seven countries (Cabo Verde, Central Africa Republic, Equatorial Guinea, Gabon, Guinea Bissau, Mali, Sao Tome and Principe). According to the WHPCA, Sao Tome and Principe, Equatorial Guinea and Gabon are developing some capacity-building initiatives, such as targeted conferences, training in palliative care, and lobbying of policymakers[7]. However, these still appear to be in the planning phase at the moment of writing this thesis.

Table 8 –Training providers per stakeholders' categories

	APCA	Other training providers
**National health authority**	Cameroon, DRC, Ghana, Liberia, Nigeria, The Gambia, Togo (7/24)	n/a
**Healthcare professionals**	Cameroon, DRC, Ghana, Liberia, Nigeria, The Gambia, Togo (7/24)	**Benin** (Association Béninoise de Soins Palliatifs)<br>**Cameroon** (Hospice & Palliative Care Association of Cameroon)<br>**Ghana** (Korle Bu Palliative Care Team)<br>**Nigeria** (Society of the Study of Pain Nigeria; Centre for Palliative Care; Attmadu Bello University Teaching Hospital)<br>**Senegal** (Alliance Pour Les Soins Palliatifs au Senegal (ASPASENI)<br>**Togo** (NGO ORJEDEC)
**Other stakeholders (medical and non-medical)**	Cameroon, Côte d'Ivoire, DRC, Ghana, Liberia, Nigeria, Senegal, Sierra Leone, The Gambia, Togo (10/24)	**Cameroon** (Hospice & Palliative Care Association of Cameroon)<br>**Ghana** (Komfo Anokye Teaching Hospital; Ghana Palliative Care Association)
**Patients and civil society**	Cameroon, DRC, Liberia, Nigeria, The Gambia, Togo (6/24)	**Nigeria** (Society of the Study of Pain Nigeria)<br>**Senegal** (Alliance Pour Les Soins Palliatifs au Senegal (ASPASEN)<br>**Guinea** (ONG Soins palliatifs de Guinée (SOPAG)

n/a = not available
a. APCA. Summary of Achievement 2011-2020
b. APCA. Annual Report 2017-2018 – Building bridges.
c. APCA. Annual Report 2018-2019 - Towards Universal Health Coverage.
d. APCA. Accelerating palliative care development in West Africa. Interest group meeting, 5th International African palliative care conferenc

Educational interventions on palliative care and opioid analgesics

The following sections describe the findings on existing educational interventions focused on the key-actors, that can influence (or have the potential to influence) the access to palliative care and opioid analgesics, these including: a) national authorities; b) healthcare professionals; c) other (medical and non-medical) stakeholders; and d) patients and civil society.

a) National authorities

In this category, we considered:

- Ministries of Health;
- regulatory authorities;
- national procurement centres;
- competent national authorities responsible for health data collection and sharing.

The APCA appears being the main training provider working with national authorities (table 8). In seven out of 24 countries, the APCA supported and coordinated several activities at the Ministry of Health level, working with: deputy ministers, key-officers, regional commissions, and local political leaders. The main activities are presented as follows: palliative care awareness raising; technical assistance and financial support (i.e.: assistance to implementing the 2014 WHA resolution on palliative care); and actions to facilitate dialogue amongst palliative care stakeholders from West Africa[47-50].

In Liberia, Togo, The Gambia, Ghana, and Nigeria the APCA implemented training activities to improving access to controlled medicines and/or to setting up a local production of affordable oral liquid morphine[48,49]. The APCA worked with relevant stakeholders, with these changing from country to country and including: the chief pharmacist in the Ministry of Health, stakeholders from the pharmaceutical supply chain (Ghana, Nigeria), the director of the Central Medical Stores (The Gambia and Liberia), the Central Purchasing Center for Essential Generic Medicines and the pharmacy directorate (Togo).
The APCA provided technical assistance for activities such as the opioids' ordering and production process, production site assessments, product quality assurance, selection of appropriate formulations, access to raw materials, product distribution, and reporting of consumption[48]. As a result, the government of Togo secured a budget for procuring morphine powder and started local morphine production in the national referral hospital[48,49]. In Ghana, the APCA offered Continuing Medical Education, both to support morphine production, and to improve data collection systems[48].
In Nigeria, the APCA collaborated with Hospice Africa Uganda (HAU) to deliver a training workshop on Good Manufacturing Practice (GMP) to staff members in the Food and Drug Administration of Nigeria's Federal Ministry of Health (NAFDAC) to increase the local morphine production[48].

In Nigeria, a training program addressed to national authorities (including the Ministry of Health, the national regulatory agency, pharmacy departments and national procurement departments) and healthcare workers has been implemented by the Treat the Pain Group (American Cancer Society), with the aim to improve access to oral morphine[51-53]. This program (Treat the Pain - the Morphine framework) required both political and clinical participation and included all the stakeholders involved in the morphine procurement and supply[51-53]. Thanks to the Treat the Pain collaboration, four teaching hospitals started to locally produce low-cost oral morphine solution in 2012. The premises producing oral morphine increased to 26 in one year, and by 2017 one federal manufacturing facility started its own production. The government's Central Medical Store distributes the oral solution to the lower level hospitals. It is estimated that 22 out of the 32 tertiary hospitals in Nigeria have procured morphine powder from the Central Medical Stores for their oral morphine production units[51-53]. However, even with recent improvements, opioids' consumption in Nigeria is still less than 0.1 mg per capita[40] and the quota requested for 2021 was 66 kg[41] (table 6), despite the estimated need for morphine in Nigeria being nearly 1,100 kg per year[53].

b) Healthcare professionals

The APCA appears to be the leading training provider also for healthcare professionals' support (table 8). Technical assistance and guidance for strengthening and integrating palliative care in teaching programmes and relevant curricula have been offered to healthcare workers, including doctors, nurses, pharmacists, social workers, dentists, anaesthetists, oncologists, and clinical officers[47-49]. The APCA delivered formal palliative care education and awareness in seven out of 24 countries, and in Togo, they also offered specific training on opioid prescription and delivery. No outcome on improved access has been reported.

In 2016 the APCA co-promoted the Project Extension for Community Healthcare Outcomes-Palliative Care in Africa, an international program aiming to increase knowledge in palliative care in hospitals' healthcare workers[54]. The program was implemented in six African countries, including Ghana and Nigeria from our sample, and rolled on for 16 months. Participants were invited to monthly online lectures. At the end of the training, doctors increased their knowledge on the appropriate use of opioid analgesics for persistent pain (from 73% at baseline to 92%, p=ns), on titrating opioids to optimize pain control (from 46% to 87%, $p<0.05$) and on managing drowsiness and other side effects (44% to 70%, p=ns). The training had no impact on other aspects of opioid management, such as dose conversion from oral to parenteral opioids and on recognizing and treating opioid withdrawal symptoms[54].

Along with APCA, eight national palliative care associations promoted education, training, research and development in pain management (table 8), but no detail on the related content and results have been found. Only the Gambian national palliative care association has reported consistency in the availability of and access to morphine tablets and syrup for use in pain relief[49].

At the moment, a palliative care curriculum in medical and nursing schools, both at undergraduate and postgraduate levels, is lacking or insufficient. Only six out of 24 analysed countries have a mandatory training course on palliative care in medical schools, whilst five countries included it in nursing schools[34,55] (table 9).

Table 9 - Medical and Nursing schools with academic curriculum for palliative care

Country	Medical schools	Medical schools - PC mandatory	Medical schools - PC optional	Nursing schools	Nursing schools - PC mandatory	Nursing schools - PC optional
Benin	2	2	0	1	0	1
Burkina Faso	4	0	1	21	0	0
Cabo Verde	n/a	n/a	n/a	n/a	n/a	n/a
Cameroon	5	0	0	5	0	1
Central Africa Republic	1	0	0	3	0	0
Chad	n/a	n/a	n/a	n/a	n/a	n/a
Congo	1	0	0	n/a	n/a	n/a
Côte d'Ivoire	2	2	0	3	3	0
DRC	12	0	1	n/a	n/a	n/a
Equatorial Guinea	0	0	0	0	0	0
Gabon	1	0	0	3	0	0
Ghana	0	0	0	25	1	24
Guinea Bissau	n/a	n/a	n/a	n/a	n/a	n/a
Guinea	4	3	0	8	8	0
Liberia	1	0	0	15	0	n/a
Mali	2	0	0	n/a	n/a	n/a
Mauritania	1	0	0	5	0	0
Niger	1	1	0	n/a	n/a	n/a

Nigeria	40[b]	3[b]	n/a	236	1	n/a
Sao Tome and Principe	0	0	0	1	n/a	n/a
Senegal	5	0	0	12	0	0
Sierra Leone	1	0	0	4	0	0
The Gambia	1	1	n/a	5	5	n/a
Togo	2	0	0	4	0	0

PC = palliative care ; n/a = not available
a. APCA Atlas of Palliative Care in Africa 2017
b. Onyeka TC et al. PLoS One 2020; 15: e0243573

Thirteen studies assessed knowledge, attitudes, and practices on palliative care and medicines management among hospital healthcare workers in DRC (1 study), Senegal (1), Nigeria (10) and Cameroon (1).

For instance, the DRC counts on 12 medical schools, but no teaching curriculum on palliative care is mandatory[34]. One study evaluated palliative care knowledge among 30 doctors and 90 nurses in six hospitals in Kinshasa. It revealed that palliative care is time-consuming and challenging to apply in hospital for 70% of health professionals, mainly because of lack of medical and technical resources. As for access to opioids, more than 70% of practitioners stated that it is not easy to prescribe morphine due to families' reluctance[56].

In Senegal, the teaching curriculum in medical and nursing school does not include palliative care[34]. A survey performed in four hospitals enrolled 376 healthcare workers, including 100 doctors, 265 nurses, and 11 social workers. Only 14% of doctors and 34% of nurses received palliative care training during their studies, and only 13% of them answered correctly to at least four out of five questions on the topic. Moreover, about 83% of the doctors declared that they rarely or never prescribed oral morphine to patients; and only 5% of them had the morphine prescription pad[57].

Much more information is available for Nigeria, where nonetheless there is similar percentage of healthcare workers formally trained on palliative care, reflecting the lack of an adequate national medical or nursing academic curriculum. Currently, only three out of 40 medical schools offer palliative care courses, as a part of Anaesthesia clerkship, with an average duration of three weeks[55]. As for nursing schools, only one out of 236 has mandatory palliative care curricula[34].
In four studies, the experience and training of healthcare professionals in Nigerian tertiary teaching hospitals was investigated by means of structured questionnaires[58-61]. Among 410 doctors, only 23.3% had basic training on pain management[58]; among 119 nurses, only 18%[59]; among 49 medical interns the 49%[58] and among 110 pharmacists, none[61].
Reflecting the lack of formal training, the general knowledge on palliative care among the healthcare professionals varied according to the study's setting. Knowledge among hospital doctors and nurses was high (above 80% in both categories, with some differences if referring to acute or chronic pain[58,59,62]), generally good among medical interns (57%)[60] while only 21.1% of pharmacist had adequate general knowledge in palliative care[61].

The lack of formal training can explain the variability on opioid analgesics use in the Nigerian hospital settings, with reported mismanagement, under-prescription, and/or under-utilisation of opioid medicines. In one study, only 9.5% of 410 doctors reported to use opioids for palliative care[58]; in a second study, 69% of 340 healthcare professionals reported to use morphine for pain management and 55.7% of them to use pentazocine[63]; and in a third study, 76% of the 170 participants agreed to the use of morphine for pain management[64].

Two additional papers investigated the opioids prescription patterns in hospital settings in Nigeria[65,66]. The first study, conducted among 114 medical doctors, revealed a poor prescription rate, despite 97.3% of the physicians reported that pain was a frequent complaint in their practice. Among them, 69.5% rarely or never prescribed opioid analgesics, mainly for fear of respiratory depression (86.8%), fear of addiction (85.1%) and non-availability (28.9%)[65]. The second study, based on the review of 736 prescriptions of morphine in one University College Hospital, showed the need for more education and advocacy programmes to increase awareness among doctors about morphine prescriptions. In fact, more than 80% of prescriptions were written in the oncology and the palliative care units, only a few in the gynaecology and medical clinics and none in the labour ward. Moreover, only 1.1% of all the prescriptions conform to international guideline concerning doubling the night dose, whilst many indicated inadequate doses or intervals and wrong titration[66].

One research evaluated the impact of the Pain-Free Hospital Initiative (PFHI), a one-year hospital-based training program on pain and opioid management in Nigeria, targeting doctors, nurses, and pharmacists and designed to improve the quality of care in 15 tertiary hospitals[52,67]. The study evaluated the impact of the program for 715 healthcare workers who had followed 13 training modules on palliative care's general knowledge for one year. It appears that 19% of participants enhanced their general knowledge on pain management and reduced misconceptions concerning opioids use, abuse risk and side effects, increasing the morphine utilisation by 60.4% (from 16,8 mg at baseline to 26,9 mg at the end the training)[67].

Finally, two papers reported the results of one pilot study conducted in Cameroon, enrolling 64 nursing students on their second or third year of University[68,69]. After a 30 hours training course, their knowledge improved about how recognizing patient' needs; providing psychological and physical support to patients; communicating patient information to the wider care team. However, their engagement in medicines management results marginal, as nurses cannot generally prescribe opioids in Cameroon[68,69]. Only nurses with special training in palliative care can prescribe them, but there is no formal policy[34].

c) Other stakeholders (medical and non medical)

In this category, we considered:

- universities, research centers, institution issuing clinical guidelines, organizations designing staff development strategies, member of the national palliative care associations;
- actors involved in the pharmaceutical supply chain;
- other regulatory and law enforcement officials.

Also in this group, the main training provider was the APCA, active in 10 out of 24 countries (table 8). It facilitated the national and international dialogue amongst palliative care stakeholders; contributed to strengthening the capacity building for higher education at academic institutions and research centers; provided technical support for implementing palliative care programs and for empowering national palliative care associations[47,49,50]. Additionally, in The Gambia, the APCA specifically targeted the Drug Law Enforcement Agency, which represents the country's agency responsible for enforcing, regulating, coordinating and controlling illicit drug trafficking and abuse, to advocate the need of improving access to controlled medicines for pain relief. Barriers to the availability and accessibility of opioids for pain relief were discussed through the National Drug Enforcement Agency and the National Pharmaceutical Services[49]. No detail on content and results has been found.

The APCA in The Gambia and Togo, the Hospice & Palliative Care Association of Cameroon, and the Ghana Palliative Care Association provided training for trainers at the hospital level, involving nurses and palliative care specialists. We found no material used for the training and any outcome on opioid access.
Along with the APCA, the WHPCA provided a 'training of trainers' manual in English, French, Swahili and Portuguese, focusing on palliative care and medicines management[70]. However, we found no information on where it has been adopted.

The International Pain Policy Fellowship promoted by the Pain & Policy Studies Group, a WHO Collaborating Center at the University of Wisconsin, represents one example of how to improve access to opioids through training for future policymakers. The initiative aimed to teach health professionals, healthcare administrators, policy experts, and lawyers on how to improve availability and access to opioid analgesics for cancer and HIV/AIDS patients[71,72].
The Fellowship provides education activities on the role and function of the international drug control system and on how to become a strategic change agent for opioid availability[71,72]. The program consists of a 1-week training session at the University of Wisconsin, and of a follow-up technical assistance to the fellows for 2 years[71]. Sierra Leone benefitted from one fellowship in 2006, a year in which no oral morphine was available. The fellow initiated a collaborative effort with the Pharmacy Board of Sierra Leone and the Ministry of Health and Sanitation to import morphine sulfate powder and reconstitute it into oral solution locally. As a result, a morphine solution compounding laboratory had been set up in the only hospice of the country in 2009[71]. Nigeria benefitted of the International Pain Policy Fellowship too in 2006 and Ghana in 2014; however no outcome has been found[72].

d) Patients and civil society

The lack of palliative care services and trained staff to provide professional home care represents a challenge for patients, families, and caregivers. The APCA and some local associations promoted the culture of palliative care and pain management and offered training for care providers, including community volunteers, civil society organizations, religious leader and patients in six out of 24 countries (table 8)[47,48].
In Togo, Liberia and The Gambia this was accomplished through face-to-face meetings, entertainment programs and 16 radio talk shows[48]. The Society of the Study

of Pain Nigeria provided information brochures on the pain clinic at University College Hospital, radio programs on pain issues, and lecturers for various groups[73,74]. Unfortunately, there seems to be a scarcity of studies from the perspective of this group. One study evaluated the awareness rate in palliative care among 302 religious leader and seminarians in Nigeria, observing that just the 31.8% of them had heard of palliative care hence raising the need for engaging this category for developing an effective palliative care strategy with the civil society[75].

One study evaluated palliative and end-of-life care experiences of family caregivers and patients in Ghana, and indicated the need for more support from the health system. Caregiving is often associated with stress and anxiety for the lack of knowledge, resources, and accessibility to opioids medicines. Moreover, acquiring morphine outside the hospital was problematic and with a high risk of buying sub-standard medicines[76]. Additionally, a commentary on palliative care in Ghana emphasised the "opiophobia" as the main barrier to access and to appropriate use of opioid analgesics among patients and family members, underling how the fear for addiction led to low demand for opioids[77].

Finally, one study was performed in DRC among 48 family members of patients hospitalised for advanced illness in Kinshasa, and receiving palliative care. The main concerns among the family members were the lack of communication between doctors, nurses and patients, which seems to be the primary cause of distress. The lack of economical support, the scarcity and expensive cost of painkillers and the poor quality of care were also listed as barriers for palliative care access[78].

Discussion

The lack or limited availability of opioid analgesics remains a persisting challenge for palliative care provision in Western and Central Africa. Training and awareness actions appear to be limited, scattered and poorly coordinated, thus insufficient to overcome the barriers to access.

In 2020, the UNODC formulated strong recommendations for implementing adequate, skills-based educational programs addressing medical students, healthcare professionals, national authorities, policymakers, community leaders, caregivers, and patients[20]. However, in our research conducted in a sample of countries with a great dearth of access to pain medicines, we identified little evidence on training programs on palliative care. This seems to be due to the weakness of the education systems at the national level, the lack of programmatic plans on palliative care, and the absence of national strategies on opioid medicines procurement and management.

Overall, the retained literature for the 24 countries included in the analysis is minimal: only 28 papers, half of them referring to Nigeria. No publication has been identified for eighteen countries, whose precarious socio-political context and fragile health systems strongly suggest the presence of unmet needs of opioid analgesics and palliative care services. The literature search provided fragmented data on palliative care and opioid analgesics knowledge and training: data often come from studies with small sample sizes, anecdotal reports on local experience (mainly in hospital settings),

and few quantitative analyses. Furthermore, attitude on palliative care, knowledge, and training were primarily evaluated among hospital healthcare professionals, while patients and caregivers' seem neglected.
At policy level, only a few studies assessed the efficacy of training addressed to policymakers and national authorities, and limited interventions have been implemented to accelerate the integration of palliative care into national health systems.

Our work presents some limitations: in particular, we did not conduct a systematic review, and we considered published documents only, rather than performing a prospective qualitative field research. Nonetheless, it can provide a basis for drafting some initial recommendations. These recommendations are listed below, each of them followed by a short justification.

Recommendation 1 – Training competent national authorities, including those responsible for data collection and data sharing.

The findings from our research indicate a distortion between the demand and the supply of opioid analgesics; and they suggest that one of the main reason is the lack of knowledge and of appropriate training about the use and management of these medicines.
According to the 2018 INCB report, the 24 countries in Western and Central Africa face blatant discrepancies between demand for opioid analgesics and real needs; furthermore, data on opioids consumption were incomplete for number of countries which submitted their estimates (11 out of 24), and for quantities declared[40]. It is unlikely that the quota presented for 2021 are accurate[41]. The miscount of requirement can cause shortages, unavailability, distortion of demand, inappropriate prescriptions, impact on costs, and over all, avoidable human suffering[3,6,8].
Hence, developing the expertise at the national level to provide precise estimates and accurately assess medical needs, is crucial to improve access. In our sample of countries, the APCA offered Continuing Medical Education to improving data collection systems only for Ghana[48]. Western and Central African countries urgently need training in planning, procurement of/access to essential opioid analgesics, to ameliorate the access for palliative care medicines and pain management.

Recommendation 2 – Training competent national authorities to improve local production and guarantee a quality assurance system.

To overcome the limited availability of morphine, five out of 24 countries set up local production, mainly supported by APCA technical advice. A good example is represented by the Treat the Pain collaboration in Nigeria, which published the outcome of the project, i.e.: increased access to morphine at hospital level[51-53]. However, although this measure provides an opportunity to locally improve access, the evidence we collected does not provide any information on:

- the typology of the production and health workers involved in the manufacturing (it is not clear whether this is an industrial production or a galenic laboratory);
- the educational activities put in place to guarantee the quality of the process and the quality and safety of the product over time (only in Nigeria, staff of the regulatory agency received a training workshop on Good Manufacturing Practice[48]);
- the plans for a continuing education system for everyone involved in the

production, production oversight, and distribution chain.

Recommendation 3 - Integration of palliative care, including opioids management, into the academic curricula; and integration of palliative care into healthcare services.

Another critical aspect leading to limited opioid analgesics availability is an underdeveloped and unevenly distributed palliative care system in Western and Central Africa[76]. Our research underlined the lack of an integrated palliative care system in all the countries in our sample, with lack of coordinated, stand-alone national palliative care plans, lack of (adequate) national legislations and lack of dedicated budget (with partial exceptions)[7,34]. The insufficient palliative care provisions and a weak palliative care system lead to an unequal quality of services and to different availability of medicines, creating further in-country inequalities.

Furthermore, all analysed countries have a serious dearth of healthcare professionals, and the lack of formal education in palliative care leads to misconceptions, and underuse of opioids. The frequent misunderstanding of palliative care among healthcare professionals limits both the palliative care referrals and the morphine prescription. Few studies evaluating the impact of training on opioid management and access demonstrated a positive effect on opioids prescription; however, they are limited to tertiary hospital settings, and they do not propose recommendations to promote a change at a broader level. Additionally, we found no publication on training contents, and we found no publication reporting long-term outcomes from the training.

Recommendation. 4 – Advocate for task-shifting across medical professions and design appropriate training for nurses and health officers.

Despite the shortage of health workforce in Western and Central Africa (less than four doctors for 10,000 inhabitants in 22 countries), opioids prescription is often restricted to medical specialists (i.e.: oncologist). As long as doctors or specialists are the only professionals legally permitted to prescribe morphine, it will be impossible to reach most of the population in need of palliative care medicines[79]. Hence, changing existing legislation to allow some doctors' roles to be 'shifted' to specially-trained nurses or clinical officers seems necessary[34,79]. Such approach has been successfully implemented to facilitate access to HIV medicines, and it started to increase access to opioids in Uganda[3,80]. In our sample of countries, nurses with appropriate training can prescribe morphine only in Cameroon and Sierra Leone; however, none of the countries in this study had nurse-prescribing laws. We found no papers describing the specific training that allows nurses to prescribe oral morphine legally. We found no publication on experiences of task-shifting for palliative care in Western and Central Africa, nor any policy and advocacy documents addressing this issue.

Recommendation 5 – Broaden the range of groups targeted by integrated training programs and related research

Training activities targeting stakeholders other than national authorities appear scarce, whilst the few identified ones did not report long-term outcomes. The only country where law enforcement officials were targeted by training was The Gambia[49]; however, we did not find details on the content, the aim and the results of this training activity.

Sierra Leone benefitted from one fellowship provided by an international collaboration with the University of Wisconsin, and the effect on improving access to opioid analgesics seems concrete[71]. Yet, this remains an isolated example.
Different groups of stakeholders, such as lawmakers and law enforcement bodies, have a key role in creating adequate conditions for making medicines for pain control timey available for those in need, thus they should be targeted by (integrated) training programs.
Furthermore, patients, caregivers and members of the society at a broader scale may play a key-role for advocating for palliative care service's availability, provided that adequate information and education is offered to the general public and to opinion leaders in the society. Also, increasing knowledge and awareness of palliative care in the community may help diminish stigma and reshape attitudes toward palliative care[3]. Unfortunately, from our research, there seems to be a scarcity of training and awareness-raising activities targeting these stakeholders. Only few studies evaluated civil society perception on palliative and, more in general, there seems to be a lack of consideration for this group's perspective. Nonetheless, these would be fundamental elements to increase access to pain-control therapies, reducing the stigma and correcting misconceptions on the risk of creating opioids dependence in Western and Central Africa.

Conclusion

Overall, our findings suggest that the limited training and educational activities implemented in 12 out of 24 Western and Central Africa countries mainly target healthcare professionals and some national authorities. The primary focus is on clinical practice and hospital patient care, leaving a serious training gaps for other very relevant stakeholders and functions. Gaps in training programs include, but are not limited to, the monitoring of opioids needs and consumption; the planning and management of stocks; the quality assurance of local morphine production; the need, fears and perceptions of patients and caregivers; the role of law enforcement authorities and their coordination with health authorities.
Furthermore, trainings are often scattered, not integrated in formal curricula, and they never bring together the different stakeholders that play key-roles for access to opioid medicines. The current focus on doctors and prescribers do not consider how their work and performance is strictly interconnected both upstream (i.e.: regulatory authorities, law-enforcement authorities, supply chain, society at large) and downstream (i.e.: nurses, clinical officers, patients, caregivers).
Lastly, trainings are often conducted in isolation, without sharing their detailed contents and without monitoring and measuring their middle and long-term outcomes. It remains also unclear if the contents are adequately tailored to specific contexts. This lack of (shared) information makes it very difficult, for the moment, to identify good practices to be applied widely.
A comprehensive and integrated educational approach involving all key actors across policymaking enforcement, healthcare and pharmaceutical systems, patients and caregivers and the society as a whole, is urgently needed to promote access to opioid analgesics for medical use in West and Central Africa. Hence, capacity building and more research should be implemented at any level.

References

1. INCB 2020. Celebrating 60 Years of the Single Convention on Narcotic Drugs of 1961 and 50 Years of the Convention on Psychotropic Substances of 1971 (E/INCB/2020/1/Supp.1). www.incb.org/documents/Publications/AnnualReports/AR2020/Supplement/00_ AR2020_supp_full_document.pdf (last access: 02.04.2021)

2. WHO 2011. Ensuring balance in national policies on controlled substances: guidance for availability and accessibility of controlled medicines. [Withdrawn; currently under review – see Joint Position Statement on the Decision by WHO to Withdraw Guidance Documents] http://globalpalliativecare.org/who-withdrawal-of-guidance-documents/ (last access: (last access: 21.03.2021)

3. Nchako E at al. Barriers to the availability and accessibility of controlled medicines for chronic pain in Africa. Int Health 2018; 10: 70-77.

4. Knaul FM et al. Alleviating the access abyss in palliative care and pain relief - an imperative of universal health coverage: the Lancet Commission report. Lancet 2018; 391: 1391-1454. doi: 10.1016/S0140-6736(17)32513-8. Epub 2017 Oct 12.

5. WHA 2014. Resolution 67.19: Strengthening of palliative care as a component of comprehensive care throughout the life course. https://apps.who.int/gb/ebwha/pdf_files/WHA67/A67_R19-en.pdf (last access: 21.03.2021)

6. UNODC 2018. Technical guidance: increasing access and availability of controlled medicines. www.unodc.org/documents/drug-prevention-and-treatment/UNODC_2018_technical_guidance_on_promoting_access_at_national_ level.pdf (last access: 21.03.2021)

7. The Worldwide Hospice Palliative Care Alliance. Global Atlas of Palliative Care - 2nd Edition. www.thewhpca.org/resources/global-atlas-on-end-of-life-care (last access: 21.03.2021)

8. Scholten W. Access to Controlled Medications: Barriers, Measuring Adequacy of Consumption, and Current Developments. JIED 2020; 2: 10–20. DOI: https://doi.org/10.31389/jied.59

9. Cherny. The Global Opioid Policy Initiative (GOPI) project to evaluate the availability and accessibility of opioids for the management of cancer pain in Africa, Asia, Latin America and the Caribbean, and the Middle East: introduction and methodology. Ann Oncol 2013; 24 (S11):xi7-13. doi: 10.1093/annonc/mdt498.

10. Berterame S et al. Use of and barriers to access to opioid analgesics: a worldwide, regional, and national study. Lancet 2016; 387: 1644–56.

11. Single Convention on Narcotic Drugs, 1961, as amended by the 1972 Protocol. www.incb.org/documents/Narcotic-Drugs/1961-

Convention/convention_1961_en.pdf (last access: 21.03.2021)

12. World Health Organization Model List of Essential Medicines. 21st List, 2019. www.who.int/publications/i/item/WHOMVPEMPIAU2019.06 (last access: 10.04.2021)

13. World Health Organization Model List of Essential Medicines for Children. 7th List, 2019. www.who.int/publications/i/item/WHOMVPEMPIAU201907 (last access: 10.04.2021)

14. Convention on Psychotropic Substances of 1971. www.unodc.org/unodc/en/commissions/CND/conventions.html (last access: 10.04.2021)

15. United Nations Convention against Illicit Traffic in Narcotic Drugs and Psychotropic Substances of 1988. www.unodc.org/unodc/en/commissions/CND/conventions.html (last access: 10.04.2021)

16. King NB et al. Untreated Pain, Narcotics Regulation, and Global Health Ideologies. PLoS Med 2013; 10: e1001411. doi:10.1371/journal.pmed.1001411

17. West Africa Commission on Drugs (WACD). Model drug law for West Africa - A tool for policymakers. September 2018. www.globalcommissionondrugs.org/wp-content/uploads/2018/08/WADC-MDL-EN-WEB.pdf (last access: 10.04.2021)

18. Brennan F et al. Access to Pain Management as a Human Right. Am J Public Health 2019; 109: 61–65. doi:10.2105/AJPH.2018.304743

19. Callaway MV et al. World Health Organization Public Health Model: A Roadmap for Palliative Care Development. J Pain Symptom Manage 2018; 55: S6eS13.

20. UNOCD 2020. Resolution 63/3: Promoting awareness-raising, education and training as part of a comprehensive approach to ensuring access to and the availability of internationally controlled substances for medical and scientific purposes and improving their rational use. www.unodc.org/documents/commissions/CND/Drug_Resolutions/2020-2029/2020/Resolution_63_3.pdf (last access: 21.03.2021)

21. The World Bank. Poverty and Equity Database (Last Updated:10/07/2020) https://databank.worldbank.org/source/poverty-and-equity# (last access: 21.03.2021)

22. The World Bank in Western and Central Africa. www.worldbank.org/en/region/afr/western-and-central-africa (last access: 21.03.2021)

23. The World Bank. Macro Poverty Outlook for Sub-Saharan Africa. www.worldbank.org/en/publication/macro-poverty-outlook/mpo_ssa (last access: 21.03.2021)

24. The World Bank Data - World Health Organization's Global Health Workforce Statistics. https://data.worldbank.org/indicator/SH.MED.PHYS.ZS?name_desc=true&locations=ZW (last access: 21.03.2021)

25. United Nations Development Programme Human Development Reports http://hdr.undp.org/en/content/download-data (last access: 21.03.2021)

26. Financing Global Health 2016: Development Assistance, Public and Private Health Spending for the Pursuit of Universal Health Coverage. www.healthdata.org/policy-report/financing-global-health-2016-development-assistance-public-and-private-health-spending (last access: 21.03.2021)

27. Institute for Health Metrics and Evaluation (IHME). Country Profiles. www.healthdata.org/results/country-profiles (last access: 21.03.2021)

28. Institute for Health Metrics and Evaluation (IHME). Health-related SDGs. https://vizhub.healthdata.org/sdg/(last access: 21.03.2021)

29. African Palliative Care Association (APCA). https://africanpalliativecare.org/ (last access: 21.03.2021)

30. The WorldwIAHPCde Hospice Palliative Care Alliance. https://www.thewhpca.org/ (last access: 21.03.2021)

31. International Association for Hospice and Palliative Care (IAHPC). Global Directory of Palliative Care Institutions and Organizations. https://hospicecare.com/global-directory-of-providers-organizations/search/?idregion=2 (last access: 21.03.2021)

32. World Health Organization. Health financing. Out-of-pocket payments, user fees and catastrophic expenditure. www.who.int/health_financing/topics/financial-protection/out-of-pocket-payments/en/ (last access: 13.04.2021)

33. GBD 2019 Universal Health Coverage Collaborators. Measuring universal health coverage based on an index of effective coverage of health services in 204 countries and territories, 1990–2019: a systematic analysis for the Global Burden of Disease Study 2019. Lancet 2020; 396: 1250–84.

34. African Palliative Care Association. Atlas of Palliative Care in Africa 2017. www.iccp-portal.org/system/files/resources/APCA_atlas.pdf (last access: 21.03.2021)

35. Clelland D et al. Palliative Care in Public Policy: Results from a Global Survey. Palliative Medicine Reports 2020, 1: 1. http://online.liebertpub.com/doi/10.1089/pmr.2020.0062

36. Rhee JY et al. Palliative care in Africa: a scoping review from 2005–16. Lancet Oncol 2017; 18: e522–31.

37. Clark D et al. Mapping Levels of Palliative Care Development in 198 Countries: The Situation in 2017. J Pain Symptom Manage 2020; 59: 794e807.

38. HOPE worldwide. https://www.hopeww.org/About_hopeww (last access: 13.04.2021)

39. Ajayi I et al. Palliative care research in Western Africa. Eur J Palliative Care 2014; 21: 1.

40. International Narcotics Control Board (INCB) - Estimated World Requirements for 2019. Statistics for 2017. www.incb.org/documents/Narcotic-Drugs/Technical-Publications/2019/Narcotic_Drugs_Technical_Publication_2019_web.pdf

41. INCB - Estimated world requirements of narcotic drugs in grams for 2021 (February update). www.incb.org/incb/en/narcotic-drugs/estimates/narcotic-drugs-estimates.html (last access: 13.04.2021)

42. International Narcotics Control Board (INCB) – Report 2020. www.incb.org/incb/en/publications/annual-reports/annual-report-2020.html (last access: 13.04.2021)

43. Fellah N et al. Accès aux analgésiques opioïdes pour les douleurs cancéreuses: des inégalités majeures – la situation en Afrique. Douleurs 2017; 18: 127-39.

44. WHO. Global Health Observatory data repository - indicator views WHO, 2019. https://apps.who.int/gho/data/node.imr.NCD_CCS_OralMorph?lang=en (last access: 21.03.2021)

45. O'Brien M et al. Improving access to analgesic drugs for patients with cancer in sub-Saharan Africa. Lancet Oncol 2013; 14: e176-82. doi: 10.1016/S1470-2045(12)70343-1.

46. Cleary J et al. Formulary availability and regulatory barriers to accessibility of opioids for cancer pain in Africa: a report from the Global Opioid Policy Initiative (GOPI). Ann Oncol 2013; 24 (S11): xi14–xi23. doi:10.1093/annonc/mdt499

47. African Palliative Care Association. Summary of Achievement 2011-2020. www.africanpalliativecare.org/images/stories/pdf/APCA_10-YEAR_ACHIEVEMENTS.pdf (last access: 13.04.2021)

48. African Palliative Care Association. Annual Report 2017-2018 – Building bridges. www.africanpalliativecare.org/images/stories/pdf/APCA_Annualreport2018.pdf (last access: 13.04.2021)

49. African Palliative Care Association. Annual Report 2018-2019 - Towards Universal Health Coverage. www.africanpalliativecare.org/images/stories/pdf/APCA%20Annual%20Report%

202019_final_1.pdf (last access: 13.04.2021)

50. APCA. Accelerating palliative care development in West Africa. Interest group meeting, 5th International African palliative care conference. www.africanpalliativecare.org/images/stories/pdf/Accelerating_PC_Development_in_West_Africa_Meeting_report.pdf (last access: 13.04.2021)

51. O'Brien M et al. Improving access to analgesic drugs for patients with cancer in sub-Saharan Africa. Lancet Oncol 2013; 14: e176-82. doi: 10.1016/S1470-2045(12)70343-1.

52. O'Brien M et al. Treat the Pain Program. J Pain Symptom Manage 2018; 55: S135-S139. doi: 10.1016/j.jpainsymman.2017.03.033.

53. Steedman M et al. Innovation can improve and expand aspects of end-of-life care in low- and middle-income countries. Health Aff 2014; 33: 1612-9. doi:10.1377/hlthaff.2014.0379.

54. Yennurajalingam S et al. Extension for Community Healthcare Outcomes-Palliative Care in Africa Program: Improving Access to Quality Palliative Care. J Glob Oncol 2019; 5: 1-8. doi: 10.1200/JGO.19.00128.

55. Onyeka TC et al. Project OPUS: Development and evaluation of an electronic platform for pain management education of medical undergraduates in resource-limited settings. PLoS One 2020; 15: e0243573. doi: 10.1371/journal.pone.0243573. (last access: 21.03.2021)

56. Masumbuku JL et al. Approche qualitative sur les soins palliatifs et d'accompagnement dans les pratiques médicales en République démocratique du Congo. Med Sante Trop 2014; 24: 83-8. doi: 10.1684/mst.2014.0311.

57. Hamdi H et al. Palliative Care Need and Availability in Four Referral Hospitals in Senegal: Results from a Multicomponent Assessment. J Pain Symptom Manage 2018; 55: 1122-1130. doi: 10.1016/j.jpainsymman.2017.11.034. Epub 2017 Dec 7.

58. Sanya EO et al. A survey on doctors' knowledge and attitude of treating chronic pain in three tertiary hospitals in Nigeria. Niger Med J 2014; 55: 106–110.

59. Elumelu TN et al. Knowledge of cancer pain management among nurses in a Nigerian tertiary health institution. Journal of Nursing Education and Practice 2014; 4. DOI: 10.5430/jnep.v4n4p74

60. Nnadi DC et al. Knowledge of Palliative Care Among Medical Interns in a Tertiary Health Institution in Northwestern Nigeria Indian J Palliat Care 2016; 22: 343-7. doi: 10.4103/0973-1075.185080.

61. Adisa R et al. Pharmacists' knowledge, attitude and involvement in palliative care in selected tertiary hospitals in southwestern Nigeri. BMC Palliat Care 2019; 18: 107. doi:10.1186/s12904-019-0492-8.

62. Fadare JO et al. Perception of Nurses about Palliative Care: Experience from South-West Nigeria. Ann Med Health Sci Res 2014; 4: 723-7. doi: 10.4103/2141-9248.141532.

63. Ajisegiri WS et al. Palliative care for people living with HIV/AIDS: Factors influencing healthcare workers' knowledge, attitude and practice in public health facilities, Abuja, Nigeria. PLoS One 2019; 14: e0207499. doi: 10.1371/journal.pone.0207499. eCollection 2019.

64. (b) Fadare JO et al. Healthcare workers knowledge and attitude toward palliative care in an emerging tertiary centre in South-west Nigeria. Indian J Palliat Care 2014; 20: 1-5. doi: 10.4103/0973-1075.125547.

65. Suleiman ZA et al. Opioid prescribing habits of physicians in Kwara State, Nigeria. Ghana Med J 2016; 50: 63-7. doi: 10.4314/gmj.v50i2.2.

66. Elumelu TN et al. Pattern of morphine prescription by doctors in a Nigeria tertiary hospital. Niger J Clin Pract 2012; 15: 27-9. doi: 10.4103/1119-3077.94092.

67. Adenekan AT et al. Impact of Pain Management Workshop on the Knowledge and Attitude of Healthcare Workers and Opioid Utilization in a Nigerian Teaching Hospital West Afr J Med 2019; 36: 232-238.

68. Bassah N t al. Preregistration nursing students' experiences of a palliative care course in a resource-poor setting. Int J Palliat Nurs 2018; 24: 388-397. doi: 10.12968/ijpn.2018.24.8.388.

69. Bassah N et al. A qualitative evaluation of the impact of a palliative care course on preregistration nursing students' practice in Cameroon. BMC Palliative Care 2016; 15: 37. DOI 10.1186/s12904-016-0106-7

70. The Worldwide Hospice Palliative Care Alliance. Palliative care toolkit. Trainer's Manual 2020. www.thewhpca.org/resources/item/palliative-care-toolkit-training-manual-2020 (last access: 13.04.2021)

71. Bosnjak S et al. Improving the availability and accessibility of opioids for the treatment of pain: the International Pain Policy Fellowship. Support Care Cancer 2011; 19: 1239-47. doi: 10.1007/s00520-011-1200-2. Epub 2011 Jun 3.

72. Cleary J et al. Pain and Policy Studies Group: Two Decades of Working to Address Regulatory Barriers to Improve Opioid Availability and Accessibility Around the World. J Pain Symptom Manage 2018; 55: S121-S134.

73. The International Association for the Study of Pain. Society for the Study of Pain, Nigeria. www.iasp-pain.org/Chapter/Nigeria (last access: 13.04.2021)

74. The Society of the Study of Pain. https://sspn.org.ng/presentations/ (last access: 13.04.2021)

75. Badru AI et al. Palliative care awareness amongst religious leaders and seminarians: a Nigerian study. Pan Afr Med J 2017; 28: 259. doi:10.11604/pamj.2017.28.259.14010. eCollection 2017.

76. Salifu Y et al. 'My wife is my doctor at home': A qualitative study exploring the challenges of home-based palliative care in a resource-poor setting. Palliat Med 2021; 35: 97-108. doi:10.1177/0269216320951107. Epub 2020 Sep 18

77. Fisch MJ. Palliative care education in Ghana: reflections on teaching in West Africa. J Support Oncol 2011; 9: 134-5. doi: 10.1016/j.suponc.2011.05.001.

78. Masumbuku JL et al. Perceptions and types of support coming from families caring for patients suffering from advanced illness in Kinshasa, Democratic Republic of Congo. Ann Palliat Med 2017; 6 (S1): S39-S46. doi: 10.21037/apm.2017.03.13. Epub 2017 May 8.

79. INCB 2018. Progress in ensuring adequate access to internationally controlled substances for medical and scientific purposes. www.incb.org/documents/Publications/AnnualReports/AR2018/Supplement/Supplement_E_ebook.pdf

80. Fraser BA et al. Palliative Care Development in Africa: Lessons From Uganda and Kenya. J Glob Oncol 2018; 4: 1-10. doi: 10.1200/JGO.2017.010090. Epub 2017 Jun 30.
